# Ovariectomized rat model of osteoporosis: a practical guide

**DOI:** 10.17179/excli2019-1990

**Published:** 2020-01-10

**Authors:** Nasibeh Yousefzadeh, Khosrow Kashfi, Sajad Jeddi, Asghar Ghasemi

**Affiliations:** 1 Endocrine Physiology Research Center, Research Institute for Endocrine Sciences, Shahid Beheshti University of Medical Sciences, Tehran, Iran; 2 Department of Molecular, Cellular and Biomedical Sciences, Sophie Davis School of Biomedical Education, City University of New York School of Medicine, NY, USA

**Keywords:** animal model, bone, osteoporosis, ovariectomy, rat

## Abstract

Osteoporosis affects about 200 million people worldwide and is a silent disease until a fracture occurs. Management of osteoporosis is still a challenge that warrants further studies for establishing new prevention strategies and more effective treatment modalities. For this purpose, animal models of osteoporosis are appropriate tools, of which the ovariectomized rat model is the most commonly used. The aim of this study is to provide a 4-step guideline for inducing a rat model of osteoporosis by ovariectomy (OVX): (1) selection of the rat strain, (2) choosing the appropriate age of rats at the time of OVX, (3) selection of an appropriate surgical method and verification of OVX, and (4) evaluation of OVX-induced osteoporosis. This review of literature shows that (i) Sprague-Dawley and Wistar rats are the most common strains used, both responding similarly to OVX; (ii) six months of age appears to be the best time for inducing OVX; (iii) dorsolateral skin incision is an appropriate choice for initiating OVX; and (iv) the success of OVX can be verified 1-3 weeks after surgery, following cessation of the regular estrus cycles, decreased estradiol, progesterone, and uterine weight as well as increased LH and FSH levels. Current data shows that the responses of trabecular bones of proximal tibia, lumbar vertebrae and femur to OVX are similar to those in humans; however, for short-term studies, proximal tibia is recommended. Osteoporosis in rats is verified by lower bone mineral density and lower trabecular number and thickness as well as higher trabecular separation, changes that are observed at 14, 30, and 60 days post-OVX in proximal tibia, lumbar vertebrae and femur, respectively.

## Introduction

Osteoporosis, a skeletal-metabolic disease, is characterized by low bone mineral density (BMD) and deterioration of the bone microarchitecture (Lelovas et al., 2008[65]; Bliuc et al., 2015[9]). Osteoporosis affects about 200 million people worldwide, including 34 % of women aged > 50 years (Strom et al., 2011[109]; Pavone et al., 2017[86]; Tian et al., 2017[111]). Osteoporosis is a silent disease until the subject experiences a fracture (Alswat, 2017[4]; Sözen et al., 2017[104]); ~40 % of women aged > 50 years experience an osteoporotic fracture within their lifetime (Stagi et al., 2013[106]; Sözen et al., 2017[104]). Osteoporosis-induced fractures are associated with a high economic burden and also increased morbidity and mortality; thus developing new strategies for prevention and treatment of osteoporosis represent an urgent need (Lin et al., 2015[72]; Pavone et al., 2017[86]). 

Animal models of osteoporosis are suitable tools for studying new prevention and treatment modalities. The first choice, and the one most commonly employed for such studies, is the ovariectomized rat model (Turner, 2001[112]). According to the Food and Drug Administration guidelines, the rat model of osteoporosis is an excellent preclinical model for postmenopausal osteoporosis (Food and Drug Administration, 1994[30]). The ovariectomized rat model of osteoporosis mimics the estrogen deficiency-induced bone loss and shows clinical manifestations of postmenopausal osteoporosis (Jee and Yao, 2001[43]; Kimmel, 2001[55]). In addition, several therapeutic agents (e.g. estrogen and bisphosphonates) currently available for managing osteoporosis have been examined in the ovariectomized rat model (Hornby et al., 2003[40]; Kavuncu et al., 2003[50]; Lelovas et al., 2008[65]).

New strategies have been assessed in the laboratory for treating osteoporosis, however, their implementation in the clinical settings has been inadequate. Such failure can be partly attributed to a number of factors, including differences in selection of the skeletal sites, age of rats at the time of ovariectomy (OVX), and variations in the duration of the OVX (Liu et al., 2015[74]). In addition, there is no standardized protocol for performing and verifying OVX with the subsequent determination of OVX-induced osteoporosis (Francisco et al., 2011[31]). The aim of this study is to provide a practical guide for establishing a rat model of osteoporosis using OVX. Advantages and disadvantages of the model are presented and points related to human studies are highlighted.

## Bone: A brief Overview

Similar to humans, bone tissue in an adult rat is composed of ~60 % minerals (95 % calcium and phosphorus as hydroxyapatite crystals) and ~40 % organic component (90 % collagen type I) (Dogan and Posaci, 2002[23]; Rai et al., 2005[89]; Feng, 2009[27]; Kenkre and Bassett, 2018[53]). Bone is a living and highly specialized connective tissue that serves a variety of functions (Taichman, 2005[110]; Clarke, 2008[14]) including production of blood cells, a role in locomotion, protection of vital organs, homeostasis of calcium and phosphate, and regulation of acid-base balance (Taichman, 2005[110]; Clarke, 2008[14]). 

There are two major types of bone: Cortical (compact) bone and trabecular (cancellous or spongy) bone (Clarke, 2008[14]; Stagi et al., 2013) that contribute mainly to mechanical and metabolic functions, respectively (Stagi et al., 2013[106]). Diaphysis regions of the long bones are cortical bone (~80 % of bones) and the inner surface of flat bones and the end of long bones are trabecular bones (~20 % of bones) (Riggs and Melton, 1986[91]; Stagi et al., 2013[106]). 

Bones of rats are similar to those of humans, representing a dynamic tissue, which is constructed and reconstructed throughout the life by bone modeling and remodeling (Karsenty, 2017[49]; Kenkre and Bassett, 2018[53]). In the trabecular bones of rats, after decreasing growth-related bone modeling, remodeling becomes the main bone activity (Chow et al., 1993[13]; Erben, 1996[25]). This transition is associated with decreased longitudinal bone growth to very low rates (Jee and Yao, 2001[43]). Trabecular bone transition occurs at month 3 in the lumbar vertebrae, and between months 6-9 in the proximal tibia metaphysis (Jee and Yao, 2001[43]). The duration of the remodeling cycles (includes four sequential phases of activation, resorption, reversal, and formation) in normal trabecular bones is ~200 days in humans and ~6 days in rats (Vignery and Baron, 1980[115]; Eriksen, 2010[26]).

## Osteoporosis

According to the World Health Organization criteria, osteoporosis is defined as a BMD that ranks 2.5 standard deviation below the mean for young healthy subjects (a T-score of ≤ -2.5 standard deviations) (WHO, 1994[120]; WHO Scientific Group on Prevention and Management of Osteoporosis, 2003[119]). Two categories of osteoporosis have been identified, primary and secondary. Primary osteoporosis is the most common form and includes postmenopausal and age-related osteoporosis (Komori, 2015[59]). Age-related osteoporosis most frequently affects cortical bone and induces somewhat gradual bone loss due to reduced osteoblast activity, whereas, postmenopausal osteoporosis most frequently affects the trabecular bones and induces a relatively rapid bone loss due to increased osteoclast activity (Riggs and Melton, 1986[91]; Duque and Troen, 2008[24]). 

In the 1940s, Albright et al. reported that estrogen could prevent osteoporosis and in the 1960s, the association between menopause and osteoporosis was identified for the first time (Ji and Yu, 2015[44]). Women lose about 50 % and 35 % of their trabecular and cortical bones, respectively throughout their life (Hunter and Sambrook, 2000[42]). Biphasic patterns of bone loss are reported for both humans and rats (Kalu, 1991[48]); the slow phase of bone loss starts at age 40 years and the rapid phase occurs in the first 2 years after menopause (Genant et al., 1982[35]; Riggs and Melton, 1986[91]; Riggs et al., 1986[92]), with the increased rate of bone loss stabilizing approximately 10 years after menopause and merging thereafter into a continuous age-related bone loss (Hunter and Sambrook, 2000[42]).

## Ovariectomized Rat Model of Osteoporosis

**Figure 1:**
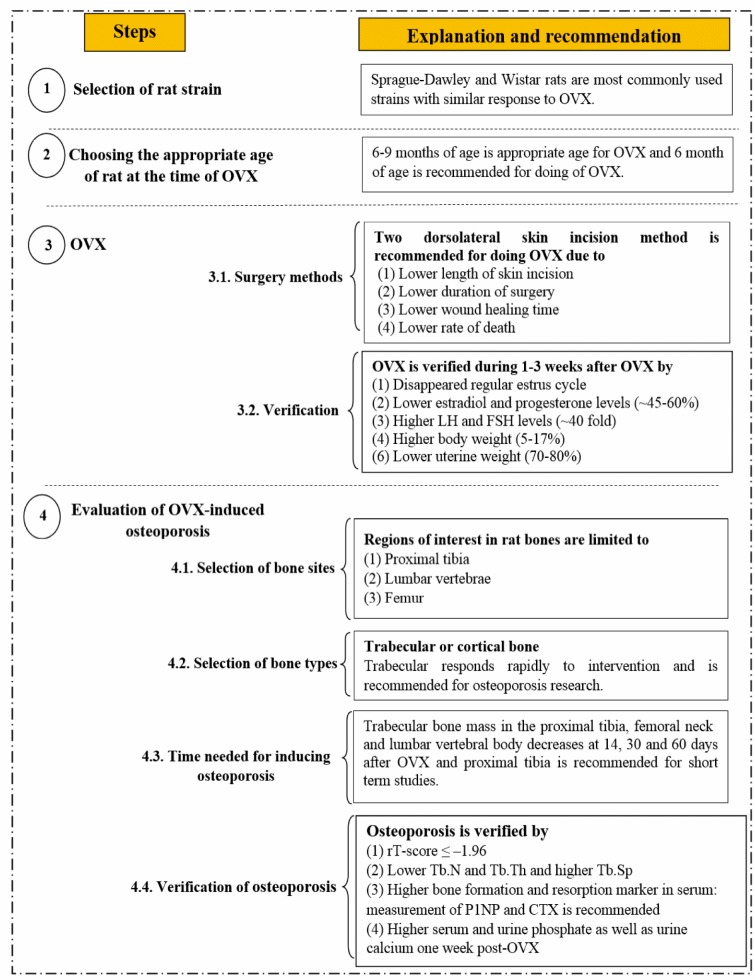
Step-by-step guideline for inducing osteoporosis using an ovariectomized rat model. OVX, ovariectomy; LH, luteinizing hormone; FSH, follicle stimulating hormone; BMD, bone mineral density; Tb.N, trabecular number; Tb.Sp, trabecular separation; Tb.Th, trabecular thickness; P1NP, amino-terminal propeptides of procollagen type I; CTX, carboxy-terminal cross-linking telopeptide of type I collagen.

Rats often do not become menopausal until 18-24 months of age, at which time they are termed aged (Brooks et al., 2016[10]); therefore rats do not experience a natural menopausal (Turner, 2001[112]). OVX is used for induction of menopause in rats (Turner, 2001[112]). In this study, we present a 4-step guideline for inducing a rat model of osteoporosis by OVX. As shown in Figure 1(Fig. 1), this guideline provides a practical guide in 4 sequential steps: (1) selection of the rat strain, (2) choosing the appropriate age of rat at the time of OVX, (3) selection of surgery method and verification of the success of the OVX, and (4) evaluation of OVX-induced osteoporosis (selection of bone sites, bone type, time needed for inducing osteoporosis) and lastly verification of osteoporosis in ovariectomized rats.

### Step 1: Selection of the rat strain 

Considerable variations exist in bone structure, BMD, and fragility phenotypes amongst Sprague-Dawley, Wistar, Brown Norway, Fischer 344, Lewis, and Wistar Kyoto rats (Turner et al., 2001[113]). Although there is no head-to-head study to compare the effects of OVX on bone parameters in different rat strains, Sprague-Dawley and Wistar rats are the most commonly used strains and show similar responses to OVX (Langdahl et al., 2016[63]). In addition, FDA guidelines do not specify which strain of rat is most appropriate for osteoporosis studies (Hornby et al., 2003[40]). Therefore, further studies are warranted to address this issue.

### Step 2: Choosing the appropriate age of rat at the time of OVX

Age of female rats plays a key role for model standardization at the time of OVX (Francisco et al., 2011[31]). Rats reach sexual and skeletal maturity at around 2.5 and 10 months of age, respectively (Jee and Yao, 2001[43]; Liu et al., 2015[74]; Jiang et al., 2018[46]). Rats aged 2-11 months that commonly undergo OVX, can be divided into three groups, i.e. <6, 6-9 and >9 months (Kalu, 1991[48]; Grynpas et al., 2000[37]).

Rats < 6 months of age have some major drawbacks that limit their usefulness for osteoporosis research. For example, in this age group, the rate of longitudinal bone growth in the proximal tibia, caudal vertebrae and in the proximal femur is about 29.5, 4.10 (Erben, 1996[25]) and 5.5 µm per day, respectively (Li et al., 1991[70]; Li and Jee, 1991[71]; Li et al., 1997[72]). In response to OVX, adverse effects such as increased trabecular connectivity have been observed in 3 months-old rats that may be due to faster growth rate in the young, compared to the old rats (Francisco et al., 2011[31]). In addition, rats aged < 6 months have higher bone loss after OVX, lower the Haversian and trabecular bone remodeling and lower sensitivity of lumbar vertebrae than long bones, characteristics which are confounding effects in these rats (Wronski et al., 1989[123]; Liu and Kalu, 1990[73]; Liu et al., 2015[74]).

In rats aged 6-9 months, skeletal growth decreases (Kalu, 1991[48]; Johnston and Ward, 2015[47]) and reaches 3.3, 1.09 (Erben, 1996[25]) and < 1 µm per day in proximal tibia, caudal vertebrae, and proximal femur, respectively (Li et al., 1991[70]; Li and Jee, 1991[71]; Li et al., 1997[69]). It has been suggested that the minimum age of rats for osteoporosis studies should be 6 months, unless one is assessing skeletal growth (Gasser and Willnecker, 2012[34]). The 6‐months old rats show the best osteoporotic response (lower trabecular connectivity and higher changes of plate-like to rod-like trabecular bone) compared to 3‐months or 10‐months‐old rats (Francisco et al., 2011[31]). In addition, rats at 6-9 months of age have a stable level of bone turnover markers in serum and urine (Sims et al., 1996[102]). Confounding effects of longitudinal bone growth and aging that are observed in rats < 6 and > 9 months, respectively are not observed in rats aged 6-9 months (Liu et al., 2015[74]). 

Rats aged > 9 months are not appropriate for the ovariectomized model of osteoporosis. As their responses to OVX and administrated drugs are slow and the study time would be much longer resulting in high costs (Grynpas et al., 2000[37]). Further, the osteoporosis effect that occurs after OVX, may be due to the aging process rather than the OVX per se (Francisco et al., 2011[31]). In addition, unlike humans, low response of the cortical bone compared with trabecular bone has been reported in rats aged > 9 months. 

To sum up, according to the literature available and summary shown in Table 1(Tab. 1) (References in Table 1: Kalu, 1991[48]; Grynpas et al., 2000[37]; Jee and Yao, 2001[43]; Laib et al., 2001[62]; Johnston and Ward, 2015[47]; Liu et al., 2015[74]; Jiang et al., 2018[46]), 6-9 months-old rats are recommended for their appropriate age for osteoporosis research with the 6-months-old being preferred to the 9 months-old rats, due to lower age-related changes (Laib et al., 2001[62]).

### Step 3: Ovariectomy 

There are several surgical methods for performing OVX in rats, as well as various parameters for verification of OVX after surgery that are discussed below.

#### Surgical methods for OVX

Prior to surgery, fasted rats (6-12 hours) are anesthetized by ketamine/xylazine or isoflurane (Ström et al., 2012[108]; Sankar et al., 2014[95]; Rigalli and Di Loreto, 2016[90]); skin area is then shaved (by electric clippers), washed (using chlorhexidine scrub and ethanol 70 %) and disinfected (by povidone iodine) (Ström et al., 2012[108]; Rigalli and Di Loreto, 2016[90]). Surgery should be done on a warmed pad (30-35^ °^C) for preventing hypothermia (Olson and Bruce, 1986[82]). Gel for eye protection is also used (Olson and Bruce, 1986[82]).

OVX in rats is done by ventral or dorsal skin incisions. In the ventral method, skin incision is made by the single transverse lateral- (Sankar et al., 2014[95]) or the single longitudinal-incision (Popović et al., 2016[88]). Duration of surgery and wound healing time are short (< 10 min and < 9 days, respectively) (Saadat Parhizkar and Latiff, 2008[94]; Khajuria et al., 2012[54]; Sankar et al., 2014[95]; Popović et al., 2016[88]; Rigalli and Di Loreto, 2016[90]), however, the gastrointestinal tract is manipulated (Lasota and Danowska-Klonowska, 2004[64]) and death rate in the first 24 h post-surgery is high (30 %) (Bazzigaluppi et al., 2018[7]), which is why this procedure is not recommended. In the dorsal method, skin is opened by a single midline (Olson and Bruce, 1986[82]), two dorsolateral (Lasota and Danowska-Klonowska, 2004[64]), or a single dorsolateral incision (Saadat Parhizkar and Latiff, 2008[94]). Two dorsolateral skin incisions are mostly recommended, because there is no need to suture the muscle; in addition, compared with a single midline incision, the skin incision is shorter in length (1-1.5 vs. 1-3 cm), duration of surgery (< 10 vs. > 15 min), and the wound healing time (9-10 vs. 10-14 days) (Lasota and Danowska-Klonowska, 2004[64]; Park et al., 2010[85]; Khajuria et al., 2012[54]). 

After surgery, the ovariectomized rats are housed individually for one week to avoid possible contamination and wound reopening (Khajuria et al., 2012[54]). Administration of midazolam (0.5-2 mg/kg intramuscularly or subcutaneously every 4 to 6 hours) (Stout Steele and Bennett, 2011[107]) and gentamicin (intramuscularly 5 mg/kg for 5 days) or antibiotic powders are also suggested for reducing the risk of self-mutilation and infection of the skin after suturing (Olson and Bruce, 1986[82]; Khajuria et al., 2012[54]; Popović et al., 2016[88]). 

#### Verification of OVX

The success of OVX in rats is mostly confirmed by changes in the estrous cycle, hormonal profile (circulating concentrations of estradiol, luteinizing hormone (LH), follicle-stimulating hormone (FSH) and progesterone), body weight, as well as uterine weight (Hao et al., 2016[38]). Success of OVX is also confirmed by failure to detect ovarian tissue at necropsy and by observation of marked atrophy of the uterine horns (Wronski et al., 1987[125]; Saul et al., 2016[96]). Other parameters such as higher tail skin temperature are also observed during the first week after OVX (Kobayashi et al., 2000[57]). In addition, results from vaginal smears show that regular estrus cycle disappears within 1 week after OVX, (Li et al., 2014[68]; Lemini et al., 2015[66]); details can be found elsewhere (Becker et al., 2005[8]). 

Serum estradiol levels in rats, aged 6 months during the diestrus and proestrus stages, are 20-30 and 90 pg/mL, respectively (Butcher et al., 1974[11]; Wise and Ratner, 1980[121]; Gore et al., 2000[36]; Koebele and Bimonte-Nelson, 2016[58]). OVX decreases estradiol levels at diestrus and proestrus stages by ~46 and 50 % after 1 week, respectively (Li et al., 2014[68]; Lemini et al., 2015[66]) and by ~60 and 90 % after 3 weeks, respectively (Mosquera et al., 2015[81]). Serum progesterone level is about 5-40 ng/mL during the diestrus stage and after OVX it decreases by ~60 % in rats aged 6 months (Butcher et al., 1974[11]; Wise and Ratner, 1980[121]; Gore et al., 2000[36]; Koebele and Bimonte-Nelson, 2016[58]). In addition, during diestrus, serum LH and FSH are 150-300 and 5-25 ng/mL, respectively, levels that increase to ~2000 ng/mL after 3 weeks of OVX (Butcher et al., 1974[11]; Wise and Ratner, 1980[121]; Gore et al., 2000[36]; Koebele and Bimonte-Nelson, 2016[58]). Therefore, measurements of serum estradiol, progesterone, LH and FSH levels before OVX and also 3 weeks after OVX are recommended for verification of OVX.

Despite pair-feeding, body weight in ovariectomized rats increased by 5-17 % at 1-3 weeks post-OVX (Li et al., 1997[69]; Høegh-Andersen et al., 2004[39]; Devareddy et al., 2008[21]; Li et al., 2014[68]; Alswat, 2017[4]; Conley et al., 2017[15]; Jiang et al., 2018[46]). After OVX, the estradiol levels decline and lead to decreased uterine weight by ~70-80 % after 3-4 weeks and by ~80-85 % after 13-14 weeks (uterine weight is ~500-600 mg in rats aged 6 months) (Ke et al., 1997[51]; Devareddy et al., 2008[21]; Conley et al., 2017[15]). Therefore, measuring body weight and uterine weight 3 weeks after surgery is recommended for further verification of OVX in rats. 

### Step 4: OVX-induced osteoporosis 

Response of rat bones to OVX is dependent on the type of bone (trabecular vs. cortical), site of bone (femur, proximal tibia, and lumbar vertebrae) and time after OVX (duration of estrogen deficiency) (Zhang et al., 2007[129]; Johnston and Ward, 2015[47]). Osteoporosis in the ovariectomized rats is confirmed by several methods including measurements of bone density, microarchitecture and bone turnover markers (BTMs) as discussed below.

### Selection of bone sites

Effects of OVX on the bones are not uniform across bone sites (Francisco et al., 2011[31]); in addition, OVX does not induce bone loss in some sites such as distal tibia metaphysis and caudal vertebrae (Ma et al., 1994[76]; Westerlind et al., 1997[118]; Jee and Yao, 2001[43]). It has been reported that 36 weeks after OVX, bone loss is higher (~57-64 %) in long bones (including humerus, ulna, distal femur, and proximal tibia) compared to spine (~57-64 %) and cranial bones (~1-3 %) (Liu et al., 2015[74]). In addition, significant bone losses in humerus, femur, tibia, and spine are observed 4 weeks post OVX, indicating that these sites are more sensitive to OVX (Liu et al., 2015[74]). Differences in the baseline rates of bone turnover, rate of longitudinal bone growth, and mechanical loading in different bones may explain observed variations in bone loss in this model of osteoporosis (Li et al., 1997[69]), suggesting that care must be taken for selecting bone sites studied, as not all sites behave in a way similar to the human (Grynpas et al., 2000[37]).

Regions of interest (ROI) in rat bones are limited to proximal tibia, lumbar vertebrae, and the femur, as these are the main sites of fracture in humans and are clinically relevant (Francisco et al., 2011[31]). Among ROI in rat bones, the proximal femur shares many histoanatomic similarities with humans (Bagi et al., 1997[6]) and OVX-induced bone loss in the proximal femur (neck of femur) is similar to that observed in hip fractures of humans (Parfitt et al., 1983[84]); not surprising then, that most researchers have focused on the proximal femur as the site of interest (Bagi et al., 1997[6]). The proximal tibia, the lumbar vertebrae, and the femur are also comparable with that of the humans due to high sensitivity to OVX (Liu et al., 2015[74]). However, these ROI in the rats show different patterns of bone loss after OVX (Wronski et al., 1988[122], 1989[124]). In addition, OVX-induced bone loss is observed earlier and is more severe in the proximal tibia than in the lumbar vertebrae or the femur (Wronski et al., 1986[126], 1989[123][124]; Francisco et al., 2011[31]), which is why, the proximal tibia is recommended for short-term studies.

### Selection of bone type 

In adult rats, different skeletal sites have different ratios of cortical to trabecular bone. The trabecular bone volumes of femoral neck, lumbar vertebral and proximal tibia are approximately 70 %, 40 %, and 30 %, respectively (Wronski et al., 1986[126]; Wronski et al., 1989[123][124]; Bagi et al., 1997[6]). In addition, cortical and trabecular bone show different patterns of bone loss after OVX, as discussed below.

#### Trabecular bone 

**Figure 2:**
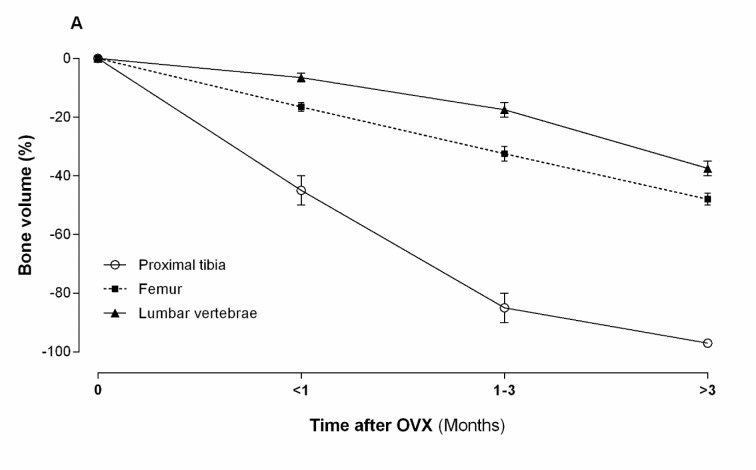
Effect of ovariectomy (OVX) on trabecular bone volume of proximal tibia, lumbar vertebrae and femur in rat

As shown in Figure 2(Fig. 2), after OVX, trabecular bone volume of proximal tibia, lumbar vertebrae and femur show different patterns of bone loss. OVX decreases trabecular bone volume in the proximal tibia by ~80 % (from 25-30 % to 5-7 %) at 90 days after OVX and by ~99 % at 540 days after OVX (Wronski et al., 1988[122], 1989[124]). OVX decreases trabecular bone volume in the femur by ~25 % between 30-90 days and by ~50 % at 180 days post OVX that remain at this level until 360 days post OVX (Li et al., 1997[69]). The trabecular bone volume in the lumbar vertebral body decreases by ~25 % (from 35-40 % to 30-35 %) during 180 days, then decreases by ~50 % between 180 and 270 days, remaining constant for 540 days (Wronski et al., 1989[123]). Therefore, compared with the proximal tibia, bone loss in the vertebrae and the femur is lower and slower after OVX (Wronski et al., 1986[126], 1989[123][124]). Decreases in trabecular bone volume in the ROI are accompanied by a deterioration of the bone microarchitecture, as observed by lower trabecular number (Tb.N) and thickness (Tb.Th) as well as higher trabecular separation (Tb.Sp) in ovariectomized rats, compared to controls (Li et al., 1997[69]). 

#### Cortical bone 

In cortical bones, OVX increases bone loss and formation in the endocortical surface and the periosteum, respectively leading to an increased and decreased bone marrow cavity and cortical thickness, respectively (Turner et al., 1987[114]; Kimmel and Wronski, 1990[56]; Miller et al., 1991[77]; Danielsen et al., 1993[16]; Aerssens et al., 1996[2]; Jee and Yao, 2001[43]; Zhang et al., 2007[129]; Komori, 2015[59]; Sharma et al., 2018[100]). Decreased thickness of the endocortical surface, adjacent to the marrow is the most sensitive and indirect index of cortical bone loss (Jee and Yao, 2001[43]; Lelovas et al., 2008[65]) and is measured by the Danielsen method (Danielsen et al., 1993[16]). Following OVX, no effect is observed on the cortical thickness until 90 days (Laib et al., 2001[62]) and the thickness of the endocortical surface adjacent to the marrow in femoral shaft decreases by only 10 % after 180 days (Danielsen et al., 1993[16]). The earliest changes in the cortical thickness and the medullary cavity size in the proximal tibia and the femoral neck are observed after 90-120 days and 360 days post OVX, respectively (Kimmel and Wronski, 1990[56]; Danielsen et al., 1993[16]; Ke et al., 1993[52]; Yamamoto et al., 1995; Li et al., 1997[69]; Zhang et al., 2007[129]; Komori, 2015[59]). Therefore, the rate of bone loss in the cortical bone is much lower and slower than the trabecular bone (Kimmel and Wronski, 1990[56]). 

To summarize, compared to cortical bone, the trabecular bone responds better and more rapidly to intervention (Grynpas et al., 2000[37]; Turner, 2001[112]) and is recommended for osteoporosis research in rats.

### Time needed for verification of OVX-induced osteoporosis

In rats, after OVX, bone resorption exceeds the bone formation leading to bone loss. Early significant bone loss in the trabecular bone of the proximal tibia, the femoral neck and the lumbar vertebral body is observed at 14, 30 and 60 days after OVX, respectively (Wronski et al., 1988[122], 1989[123][124]; Li et al., 1997[69]; Laib et al., 2001[62]). Then, bone resorption and formation reach a steady state for which the time needed varies in rats depending on the skeletal sites and is between 90-270 days (Jee and Yao, 2001[43]; Lelovas et al., 2008[65]). Bone loss reaches a steady state at ~90 days (Wronski et al., 1988[122]) for the proximal tibia, ~270 days (~39-77 weeks) for the lumbar vertebral body (Wronski et al., 1989[123]), and ~270 weeks for the femoral neck (Li et al., 1997[69]). It has been reported that the time needed for 50 % bone loss following OVX is 30-60 days for the proximal tibia 180-270 days for lumbar vertebral body and femoral neck (Wronski et al., 1989[123]; Zhang et al., 2007[129]). Therefore, according to the time needed for assessment of bone parameter after OVX, the proximal tibia is the most appropriate for short-term studies. 

### Verification of osteoporosis 

Ovariectomized-induced osteoporosis in rats is mainly confirmed by measurement of the bone density and the bone microarchitecture parameters. Measurement of bone turnover markers and biochemical parameters in serum and urine are also recommended. These methods in rats and humans are the same (Lelovas et al., 2008[65]) and are presented below. 

#### Bone density

**Figure 3:**
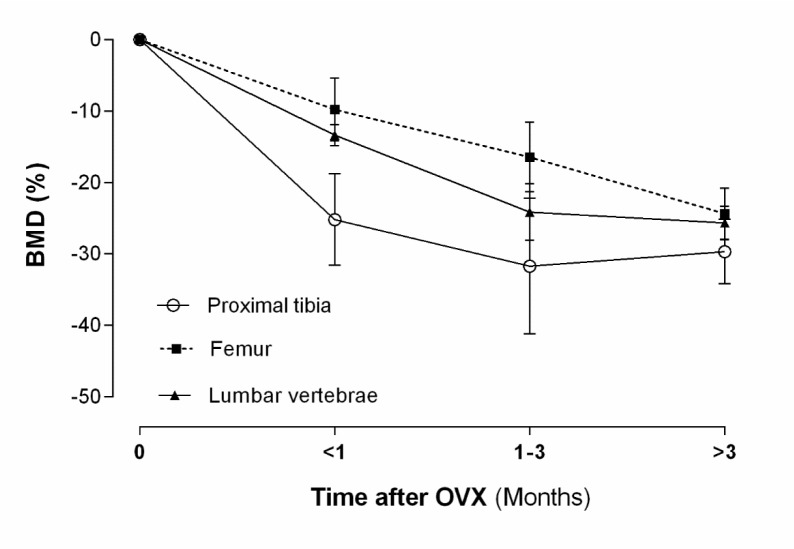
Effect of ovariectomy (OVX) on bone mineral density (BMD) of proximal tibia, lumbar vertebrae and femur in rat

Clinically, measurement of BMD is used to classify the onset and the extent of osteoporosis. As shown in Figure 3(Fig. 3), OVX decreases BMD in the proximal tibia within 30 days by ~14 % (normal level ~0.25 g/cm^2^), in the lumbar vertebrae by ~12 % (normal level ~0.25 g/cm^2^), and in the femur by ~5 % (normal level ~0.21 g/cm^2^); these values are decreased to ~40 %, ~33 % and ~34 % respectively, during 90 days after OVX. Based on human T-scores, the power of osteoporosis identification in rats is low whereas false negativity is high. Sirvastava et al. reported that the rat T-score (rT-score) has a good power for detection of osteoporosis; rT-score ≤ -1.96 indicates osteoporosis in rats (Srivastava et al., 2008[105]).

#### Method for measurement of bone mineral density

BMD is measured by dual-energy X-ray absorptiometry (DEXA), which is the gold-standard method used in clinical settings for diagnosing osteoporosis, bone fracture risk and evaluation of treatment efficacy (Osterhoff et al., 2016[83]). This technique facilitates conducting of longitudinal studies and is also used to measure lean and fat mass (Turner, 2001[112]; Ammann and Rizzoli, 2003[5]). DEXA cannot separate trabecular from cortical bone indices (Ferretti, 1995[28]; Moisio et al., 2003[78]) and over half of the fractures occurring at a BMD level are not classified as osteoporotic by DEXA (Hsu et al., 2014[41]). BMD determines only ~60-70 % of bone strength (Ammann and Rizzoli, 2003[5]); hence, by itself, it is a relatively poor predictor of osteoporosis and is not the only determinant of bone strength; therefore, other potential determinants, such as bone quality, also need to be considered that contribute to the severity of osteoporosis and bone fracture risk (Fonseca et al., 2014[29]; Rosales Rocabado et al., 2018[93]). 

#### Bone microarchitecture

Trabecular bone microarchitecture is documented as a key component of bone quality and bone strength and, thus, should also be considered in osteoporosis research (Dempster, 2000[20]; Francisco et al., 2011[31]). A variety of structural indices are presented to characterize the properties of trabecular bone, including Tb.Sp, Tb.N, and Tb.Th (Francisco et al., 2011[31]). 

**Figure 4:**
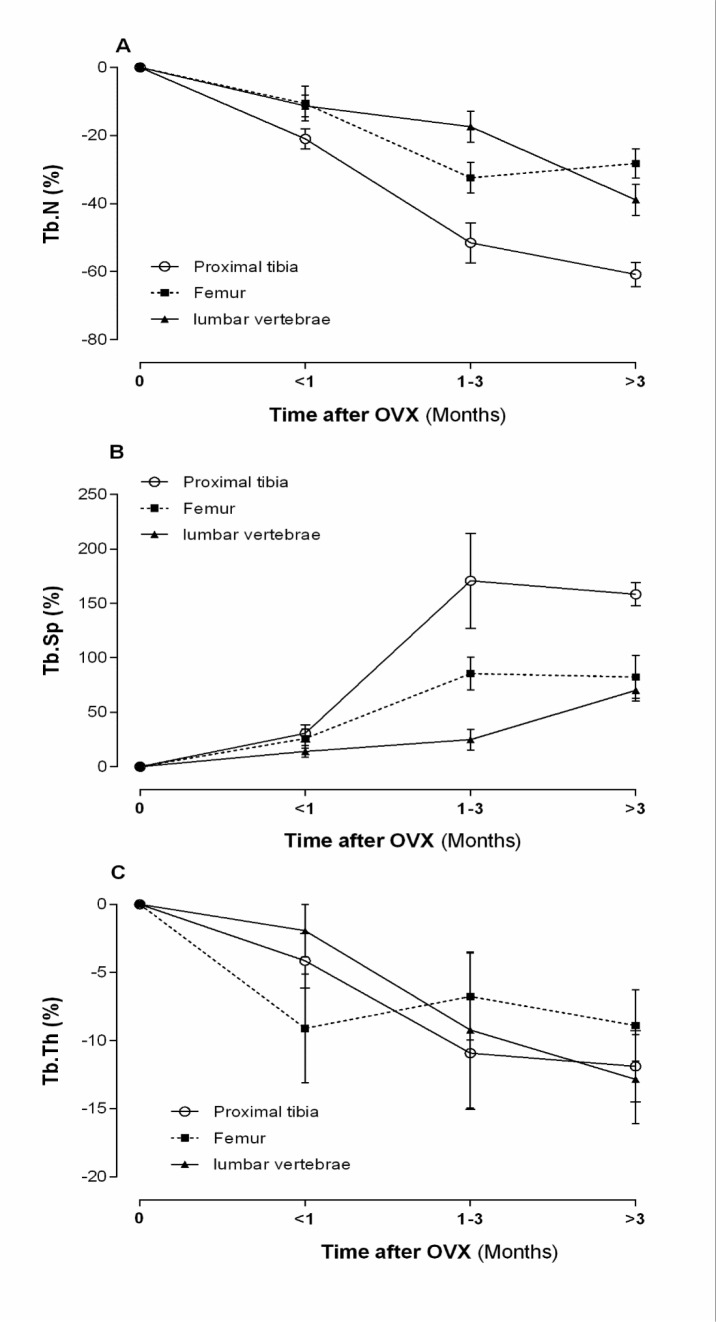
Effect of ovariectomy (OVX) on trabecular number (Tb.N) (A), trabecular separation (Tb.Sp) (B), and trabecular thickness (Tb.Th) (C) of proximal tibia, lumbar vertebrae and femur in rat

According to literature, normal levels of Tb.Sp are ~190, ~260 and ~145 µm; Tb.N are ~4.4, 3.8 and ~5.4 mm^−1^ and Tb.Th are ~80, ~100 and ~90 µm in the proximal tibia, the lumbar vertebrae and the femur, respectively. As shown in Figure 4(Fig. 4), OVX increases Tb.Sp by ~33 %, ~10 % and ~40 %, decreases Tb.N by ~22 %, ~4 % and ~19 % and decreases Tb.Th by ~7 %, ~1 % and ~8 %, in the proximal tibia, the lumbar vertebrae and the femur, respectively. 

#### Method for measurement of trabecular bone indices

Trabecular bone indices are measured by microcomputed tomography (µCT), that evaluates both geometric (cortical and trabecular indices) and densitometric (i.e. BMD) parameters of bone and is also used to describe bone quality (Krause et al., 2014[60]). The use of µCT in longitudinal studies is not recommended in some studies (Waarsing et al., 2004[116]), because of the amount of radiation exposure and also the associated costs, both of which are higher than DEXA (Dare et al., 1997[17]; Hsu et al., 2014[41]). However, Longo et al. (2016[75]) reported that longitudinal scanning of tibia by µCT with low amount of radiation exposure (low resolution) at different times within the study window (13, 17, 21 and 25 weeks after OVX) did not cause adverse effects on trabecular indices of tibia. 

#### Bone turnover markers 

Measurement of bone turnover markers (BTMs) in serum and urine, released during the formation and resorption of bone, are recommended for the verification of osteoporosis (Frolik et al., 1996[32]). Animals should be fasted for 24-36 hours before blood collection and 12 hours before 24-hour urine collection for measurements of BTMs (Sims et al., 1996[102]). Measurement of BTMs characterizes acute changes in bone turnover in the whole skeleton, however these do not provide any information regarding bone mass or bone strength; also, a number of interfering factors such as circadian rhythm, and food intake could affect BTMs (Morris et al., 1992[79]; Frolik et al., 1996[32]; Schlemmer and Hassager, 1999[97]; Seibel, 2003[99]).

Estrogen deficiency is associated with high levels of both bone-resorption and bone-formation markers (Garnero et al., 1996[33]; Charatcharoenwitthaya et al., 2007[12]). In rats, the bone formation markers are alkaline phosphatase (ALP), osteocalcin (OC), and amino-terminal propeptides of procollagen type I (P1NP). These are preferentially measured in the serum, while bone resorption markers including carboxy-terminal cross-linking telopeptide of type I collagen (CTX), pyridinoline (PYD), and deoxypyridinoline (DPD) are measured in both serum and urine (Dick et al., 1996[22]; Kuo and Chen, 2017[61]). 

According to literature, normal levels of OC, ALP and P1NP are ~20 µg/L, ~38-78 U/L and ~75 ng/mL, respectively in 6-months-old female rats. OVX increases serum OC and ALP by 20-30 % and 30-60 % after 1-6 weeks (Dick et al., 1996[22]; Schulz and Morris, 1999[98]; Davey and Morris, 2005[18]). P1NP is increased by 13 % and 106 % 12 and 18 weeks after OVX, respectively (Smith et al., 2014[103]). In addition, urine PYD increases by 35-50 % after 1-3 weeks and urine DPD increases by 35-50 % (Morris et al., 1992[79]; Sims et al., 1996[102]; Schulz and Morris, 1999[98]; Davey and Morris, 2005[18]), and 172 % during 1-3 and 12 weeks after OVX, respectively (Smith et al., 2014[103]). CTX is elevated by 74.4 % during 18 weeks after OVX (Wei et al., 2014[117]). Among these markers, P1NP (a bone formation marker) and CTX (a bone resorption marker) are the most reliable markers proposed by the International Osteoporosis Foundation (IOF) and the International Federation of Clinical Chemistry and Laboratory Medicine because of having smaller circadian variations, more stability at room temperature, and a good assay precision (Shetty et al., 2016[101]). Serum phosphate increases in ovariectomized rats from week 1 and remains elevated until week 9 (Morris et al., 1992[79]; Dick et al., 1996[22]; Sims et al., 1996[102]), then restores to normal levels at week 17 (Li et al., 2013[67]). In addition, although urine phosphate increases one week after OVX, it returns to normal levels by the third week (Dick et al., 1996[22]). Results regarding serum calcium levels are controversial, but in the most studies unchanged levels of serum calcium have been reported at 1 (Sims et al., 1996[102]), 2 (Davey and Morris, 2005[18]), and 17 weeks (Li et al., 2013[67]) post OVX. In addition, urine calcium levels are increased one week after OVX returning however to normal levels within 2-6 weeks (Morris et al., 1992[79]; Dick et al., 1996[22]).

## Potential Limitations for Using Ovariectomized Rat Model of Osteoporosis

Potential limitations and solutions to the use of ovariectomized rat model of osteoporosis are presented in Table 2(Tab. 2) (References in Table 2: Aerssens et al., 1993[1]; Dawson, 1925[19]; Grynpas et al., 2000[37]; Jee and Yao, 2001[43]; Jiang et al., 1997[45]; Lelovas et al., 2008[65]; Mosekilde et al., 1993[80]; Peng et al., 1994[87]; Turner et al., 1987[114]; Turner, 2001[112]; WHO Scientific Group on Prevention and Management of Osteoporosis, 2003[119]; Yoshitake et al., 1999[128]). Major potential drawbacks are the presence of longitudinal bone growth in mature rats after OVX, higher modeling activity, and absence of naturally fragility fracture in the rat skeleton.

## Conclusion

To conclude, although no animal model is perfect, the similarities in response to estrogen deficiency and therapeutic agents between the human and rat skeleton, have made the ovariectomized rat model an appropriate model in osteoporosis research. Current data show that the responses of the rat bones to OVX are dependent on the age of rat at the time of OVX (rats aged 6 months are more appropriate), the type of bone (trabecular bone recommended), the site of bone (proximal tibia, lumbar vertebrae and femur recommended) and the duration of OVX (14, 30 and 60 days after OVX recommended for proximal tibia, femoral neck, and lumbar vertebral body). 

Some important areas however remain to be elucidated regarding this model, including molecular mechanisms involved in OVX-induced osteoporosis. In addition, the effects of some diseases on the ovariectomized rat model of osteoporosis, and the combination effects of estrogen deficiency plus a low calcium diet, for acceleration of bone loss in rats warrant further clarification.

## Acknowledgements

The authors wish to acknowledge Ms. Niloofar Shiva for critical editing of English grammar and syntax of the manuscript. This study was supported by Shahid Beheshti University of Medical Sciences [grant no. 19198-1], Tehran, Iran.

## Conflict of interest

The authors declare that they have no competing interest.

## Author contributions

N. Y., S. J., and A. Gh. substantially contributed to conception and design of the study. N. Y., S. J., and A. Gh. and Kh. K. contributed to drafting the article or revising it critically for important intellectual content. All authors agreed on the final approval of the version to be published. 

## Figures and Tables

**Table 1:**
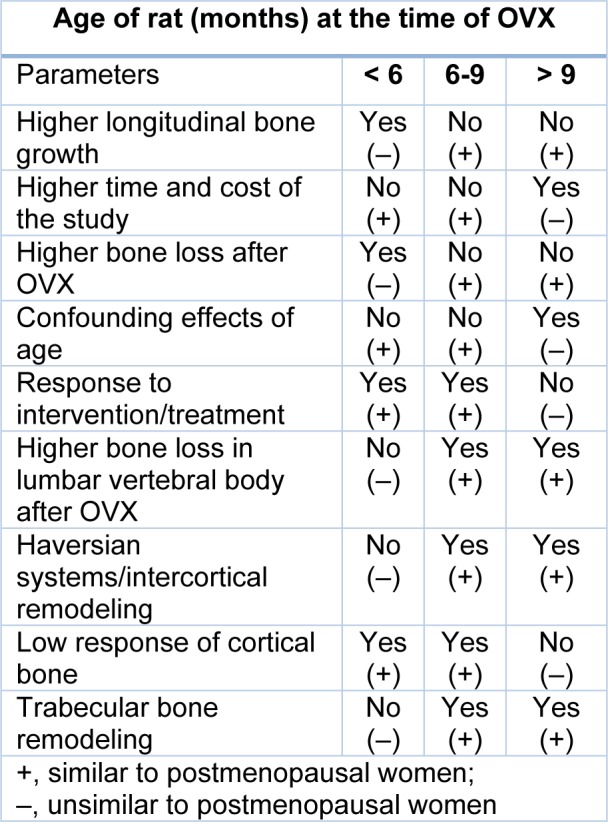
Advantages and disadvantages of OVX at different ages in rats (Kalu, 1991; Grynpas et al., 2000; Jee and Yao, 2001; Laib et al., 2001; Johnston and Ward, 2015; Liu et al., 2015; Jiang et al., 2018)

**Table 2:**
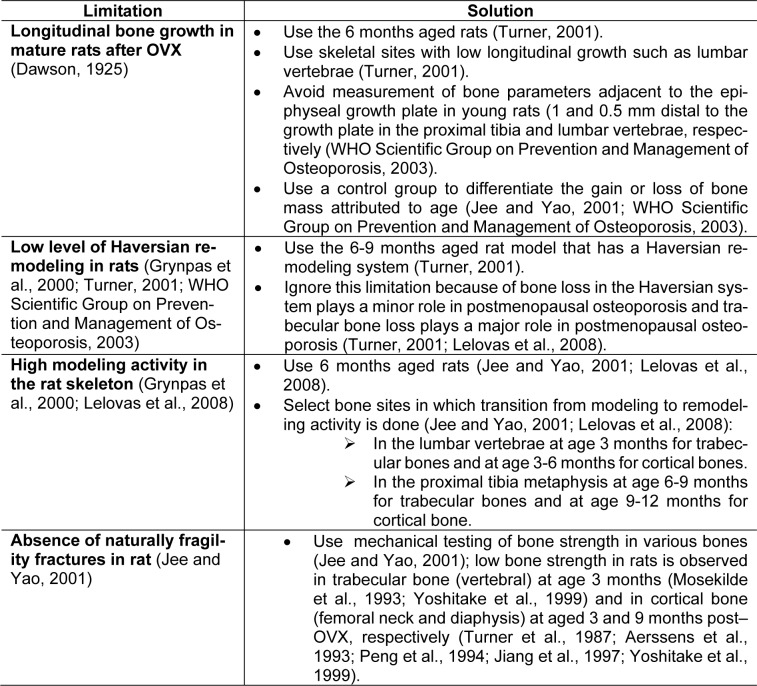
Potential limitations and proposed solutions for using the ovariectomized rat model of osteoporosis
